# Erdheim-Chester Disease Revealed by Central Positional Nystagmus: A Case Report

**DOI:** 10.3389/fneur.2022.880312

**Published:** 2022-04-07

**Authors:** Alexandra Weckel, Yohann Gallois, Rachel Debs, Bernard Escude, Laurent Tremelet, Fanny Varenne, Damien Biotti, Dominique Chauveau, Fabrice Bonneville

**Affiliations:** ^1^ENT, Department of Neurotology and Pediatric ENT, Pierre Paul Riquet Hospital, University Hospital of Toulouse, Toulouse, France; ^2^Department of Neurology, Pierre-Paul Riquet/Purpan University Hospital, Toulouse, France; ^3^Service de Radiologie, Clinique Pasteur, Toulouse, France; ^4^ENT, Clinique du Dr Honoré CAVE, Montauban, France; ^5^Department of Ophthalmology, Pierre-Paul Riquet/Purpan University Hospital, Toulouse, France; ^6^Institut Toulousain des Maladies Infectieuses et Inflammatoires (Infinity) INSERM UMR1291 - Université Toulouse III, Toulouse, France; ^7^Department of Nephrology and Organ Transplantation and Referral Center for Rare Renal Diseases, CHU Rangueil, Toulouse, France; ^8^Department of Neuroradiology, Pierre-Paul-Riquet/Purpan University Hospital, Toulouse, France

**Keywords:** Erdheim-Chester disease, dizziness, gait, nystagmus, central positional nystagmus, case report

## Abstract

Erdheim-Chester disease (ECD) is a rare histiocytic disorder, recently recognized to be neoplastic. The clinical phenotype of the disease is extremely heterogeneous, and depends on the affected organs, with the most frequently reported manifestations being bone pain, diabetes insipidus and neurological disorders including ataxia. In this article, we report on a case of a 48-year-old woman, whose initial symptom of gait instability was isolated. This was associated with positional nystagmus with central features: nystagmus occurring without latency, clinically present with only mild symptoms, and resistant to repositioning maneuvers. The cerebral MRI showed bilateral intra-orbital retro-ocular mass lesions surrounding the optic nerves and T2 hyperintensities in the pons and middle cerebellar peduncles. A subsequent CT scan of the chest abdomen and pelvis found a left “hairy kidney”, while ^18^ F-FDG PET-CT imaging disclosed symmetric 18F-FDG avidity predominant at the diametaphyseal half of both femurs. Percutaneous US-guided biopsy of perinephric infiltrates and the kidney showed infiltration by CD68(+), CD1a(-), Langerin(-), PS100(-) foamy histiocytes with *BRAF*^*V*600*E*^ mutation. The combination of the different radiological abnormalities and the result of the biopsy confirmed the diagnosis of ECD. Many clinical and radiological descriptions are available in the literature, but few authors describe vestibulo-ocular abnormalities in patients with ECD. Here, we report on a case of ECD and provide a precise description of the instability related to central positional nystagmus, which led to the diagnosis of ECD.

## Introduction

Although Erdheim-Chester disease (ECD) is a rare non-Langerhans cell histiocytic disease, over the past decade more than 1,000 cases have been reported in the literature (1). The first reports on two patients date back to 1931 (William Chester and Jakob Erdheim), but it was not until 1972 that it was named by Jaffe after he had reported a similar case (1).

Until recently, it was considered an inflammatory, non-neoplastic disease of unknown origin. However, based on recent studies, it is now acknowledged that it is a clonal myeloid disorder due to mutations that activate MAPK (mitogen-activated protein kinase *RAS-RAF-MEK-ERK*) pathways (1). In most cases there are mutually exclusive activating ERK mutations, including *BRAF*^*V*600*E*^ and MAP2K1 mutations (2).

The diagnosis of ECD is based on a combination of histopathological, clinical and radiological features. Classic solid lesions show an infiltration of typically foamy or spumous lipid-laden histiocytes with admixed or surrounding fibrosis (2). In ECD, tissues are infiltrated by foamy CD68(+), CD163(+), Factor XIIIa(+), CD1a(–), and Langerin(–) fibrotic histiocytes.

These histological impairments clinically result in systemic damage affecting the urinary tract, bone structures and central nervous system. In the latter case, dizziness is frequently reported by patients and examinations of the cerebellum are well described. However, the literature shows that adequate vestibular examination is frequently overlooked, the diagnosis of ECD being often based on extra-vestibular signs.

We are the first to describe the clinical presentation of a patient, whose only initial symptom was dizziness and isolated instability with clinical positional nystagmus.

## Case Presentation

A 48-year-old woman presented with gait instability. Her only medical history was total thyroidectomy for hyperthyroidism due to a nodular goiter in 2002.

Starting in September 2020, she began to experience dizziness, which was never really rotatory. She described unsteadiness and visual blurring during horizontal or vertical movements, with an impression that this symptom worsened over time. Given the persistence of this complaint, she consulted a doctor in December 2020. An ENT specialist identified signs of a possible lateral canal BPPV and after the 1st maneuver referred the patient to physical therapists for repositioning maneuvers. Despite 10 repositioning maneuvers, her symptoms persisted.

She was therefore admitted for consultation in our ENT department in June 2021, for diagnostic and therapeutic advices. On clinical examination a discrete static cerebellar syndrome (isolated wide base) was detected. There was no distal hypopallesthesia, deep tendon reflexes were globally present and symmetrical, with signs of pyramidal involvement (bilateral Rossolimo's sign more marked on the left side, without Babinski sign). Abdominal reflexes were normally present on the right and absent on the left. The patient also had no vesico-sphincteral disorders. There was no dysarthria, no dysmetria or dysdiadochokinesia and no extrapyramidal syndrome. She reported mild dysarthria and transient aphasia that were undetectable.

There was no ocular instability, and no spontaneous nystagmus in the light or in the dark. There was no saccadic intrusion, normal smooth pursuit and no ocular misalignment. Head shaking caused intense instability, concomitant with nystagmus with a down beat component, which was low and transient (5 s). During the supine roll test, we noted very discrete geotropic horizontal nystagmus on the left side, without any concomitant vertigo, occurring without latency, and of 30 s' duration. During the right supine roll test, we noted geotropic nystagmus, occurring without latency, and of short duration (5 s), without any concomitant vertigo. The Dix-Hallpike test was negative. Bedside or video head impulse test was not performed. With this atypical positional nystagmus (PN), a central nervous system cause was suspected. Actually, the fact that vertical downbeat nystagmus was caused by head shaking and the roll maneuver immediately triggered pure horizontal nystagmus (without a torsional component), without latency or vertigo but only dizziness, which was persistent despite 10 repositioning maneuvers, were not in favor of PN of peripheral origin.

**Figure 1:**
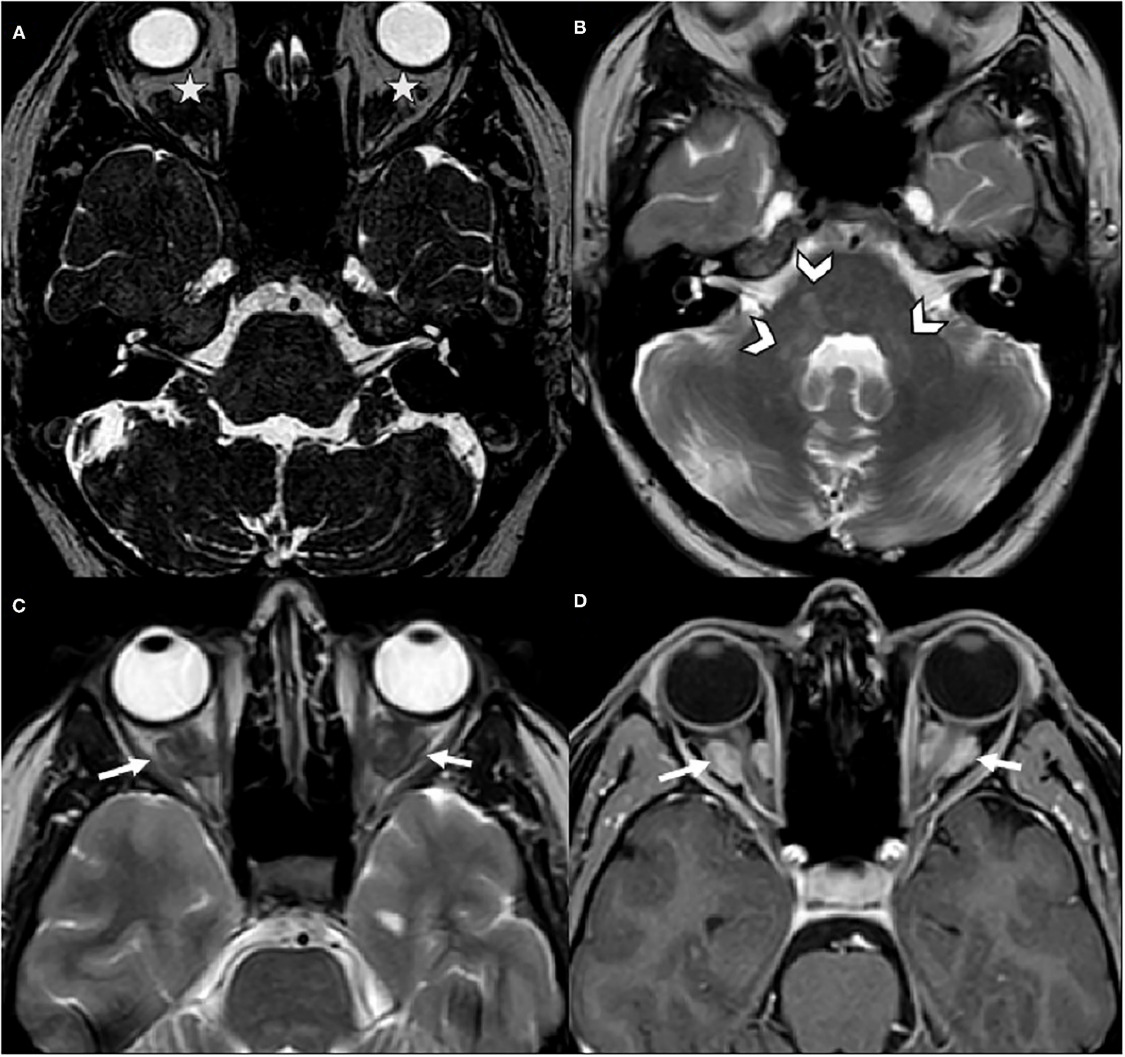
Brain MRI. **(A)** Axial CISS was normal for internal auditory canals and cerebellopontine angles, but incidentally depicted bilateral intra-orbital mass lesions (stars). **(B,C)** Axial spin echo T2-weighted images revealed hyperintensities in the pons and the middle cerebellar peduncles (arrowheads) and demonstrated bilateral hypointense retro-ocular mass lesions (arrows). **(D)** Axial post-gadolinium T1-weighted image, these lesions surrounded the optic nerves sheaths and were avidly enhanced (arrows).

Subsequently, the patient had a brain MRI (Figure 1) which showed mild T2 hyperintensities in the pons and bilateral middle cerebellar peduncles. Incidentally, it also revealed bilateral intra-orbital retro-ocular mass lesions surrounding the optic nerves. The lesions were hypointense on T2-weighted images and were avidly enhanced after gadolinium injection.

The patient also reported that she had been experiencing blurring in the left eye for a few months, after she was informed of the presence of an intra-orbital mass. A complete ophthalmologic examination was performed and was normal (Fundus, Visual Field, Visual Acuity and RNFL-OCT [Optical coherence tomography]).

The presence of these lesions led us to consider the possibility of ECD. Therefore, we performed further tests, including whole body FDG-PET/CT, blood tests and cardiac MRI, as recommended (3). She was referred to the neurology and nephrology departments.

**Figure 2:**
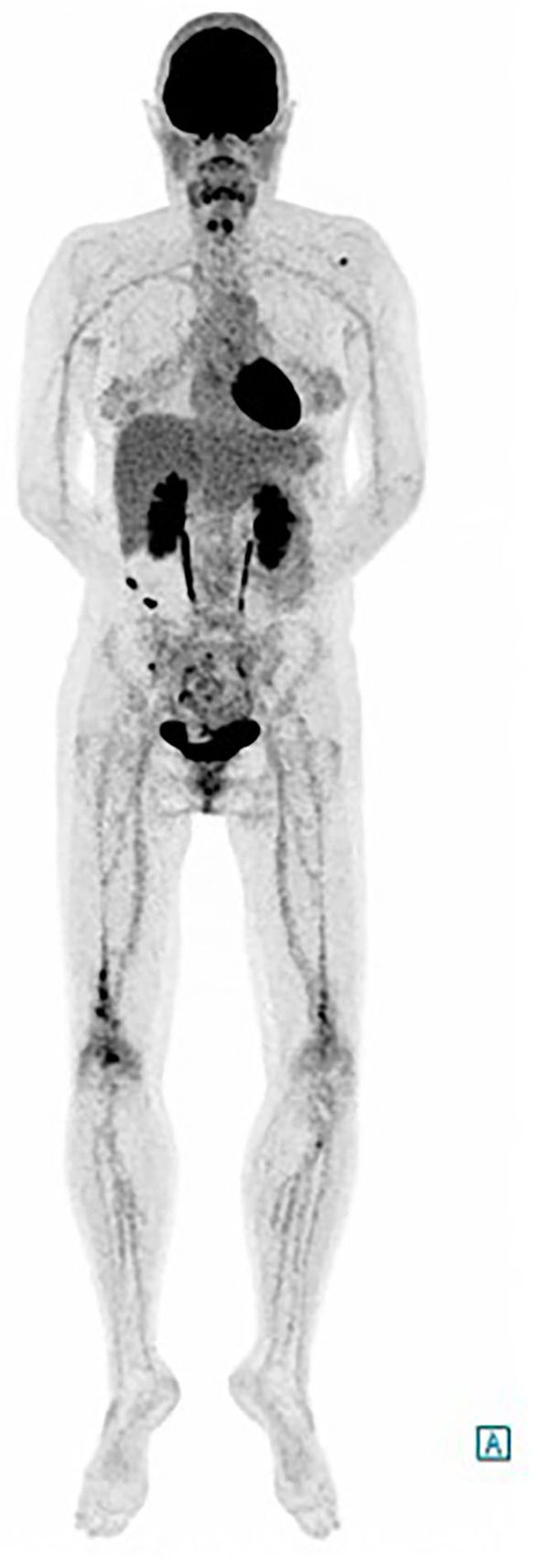
FDG-PET/CT, showing multifocal radiotracer uptake in the long bone marrow predominantly on the distal half of the femurs.

**Figure 3:**
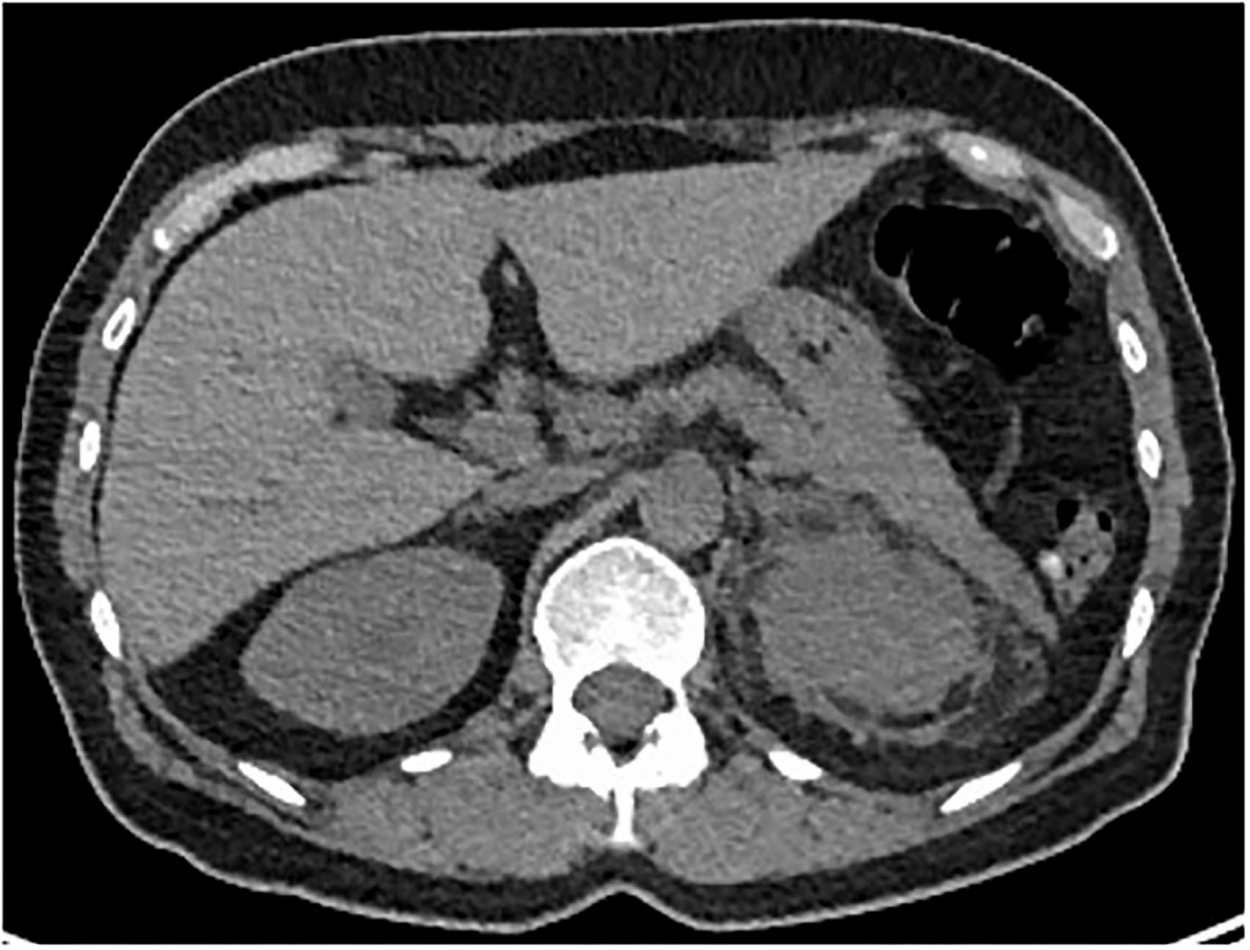
Axial CT-Scan showing dense infiltration of left perinephric adipose tissue (“hairy kidney”).

The whole body ^18^F-DFG PET/CT found multifocal radiotracer uptake in the long bone marrow, predominantly on the distal half of the femurs (Figure 2) associated with moderate radiotracer uptake of left perinephric tissue. The CT scan showed dense infiltration in upper left quadrant of the kidney (“hairy kidney”) (Figure 3). These findings confirmed the suspicion of ECD.

The cardiac MRI was free of abnormalities. Biological findings included normal renal function (S creatinine, 58 μmol/l), no diabetes insipidus, and normal CRP (C reactive protein), but thrombocytosis.

**Figure 4:**
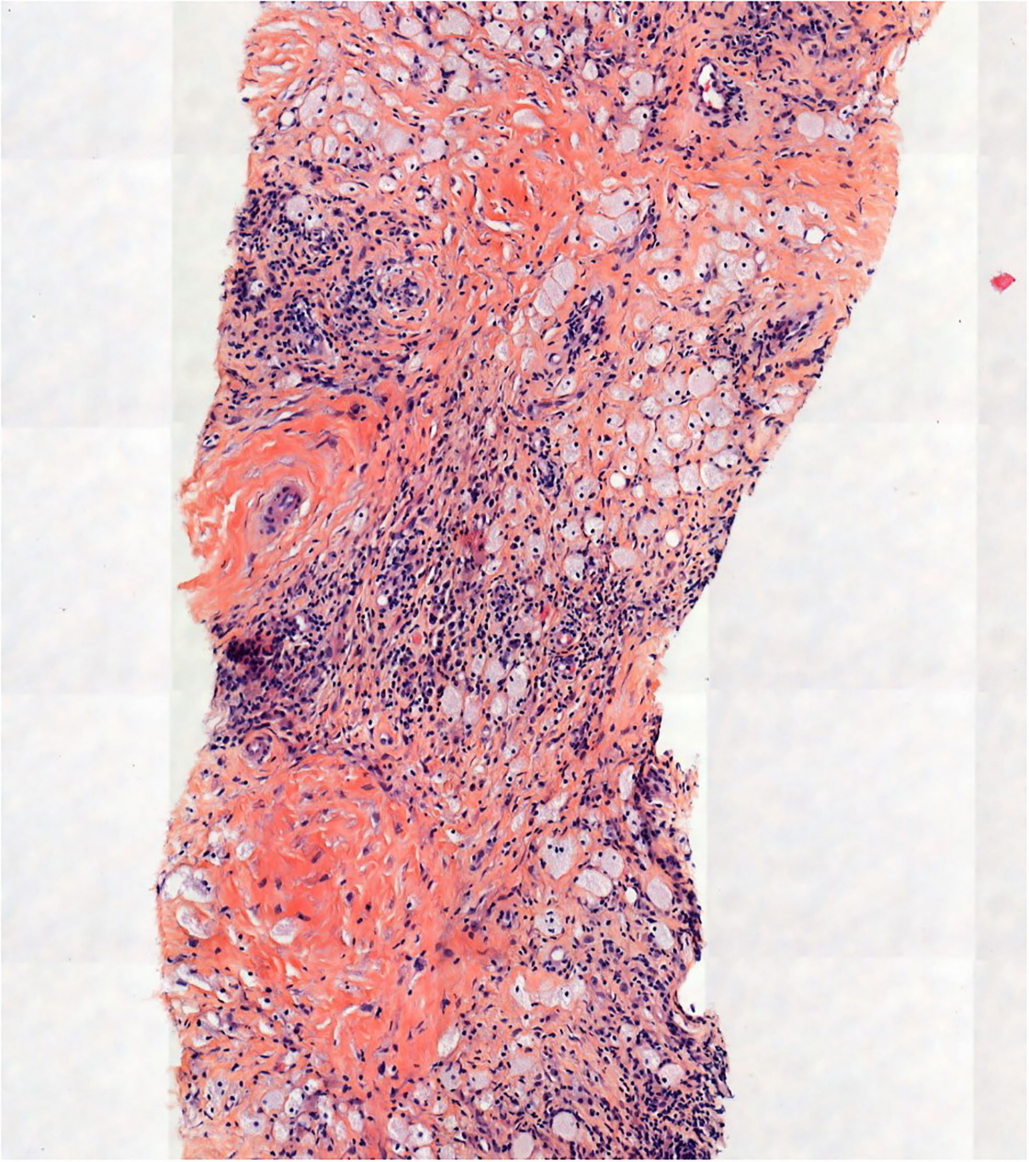
Pathological examination showed perirenal fat and kidney capsule infiltrate made up of histiocytes, some of them with foamy cytoplasm (hematein-eosin-saffron, original magnification, x100).

A left ultrasound-guided percutaneous renal biopsy of the upper and peri-renal tissue was performed. A pathological examination showed perirenal fat and kidney capsule infiltration by spumous macrophages and CD68(+), CD1a(-), Langerin(-), PS100(-) foamy histiocytes bearing the *BRAF*^*V*600*E*^ mutation (Figure 4).

Based on established diagnostic recommendations (3), ECD was confirmed based on:

- progressive neurological disorders via the brainstem, with disorders of balance, and brain MRI images
- Bone ^18^F DFG-avidity on PET/CT
- Unilateral left hairy kidney and perirenal infiltration with CD68(+), CD1a(-) foamy histiocytes bearing the *BRAF*^*V*600*E*^ mutation.

Our patient was treated as soon as the diagnostic was confirmed (end of September 2021) with vemurafenid (480 mg x 2 per day). In fact, the recommendations for first-line therapy is BRAF-inhibitor therapy with vemurafenib or dabrafenib (3). She developed numerous side effects, including severe loosening of the skin, diarrhea, red eyes, and weight loss. The treatment was first reduced (240 mg x 2 per day), then stopped after 2 months because of the persistence of these side effects. Treatment with dabrafenib was therefore initiated (75 mg x 2 per day). As of initiation, she presented iatrogenic bilateral anterior and intermediate uveitis predominantly on the right with contiguous papillitis on the right, which led to treatment cessation and reintroduction 10 days later, associated with trametinib (0.5mg x 2 per day). Besides uveitis, the ophthalmological examination remained normal.

Except for side effects, clinically the patient is perfectly stable. The patient continues to complain of isolated instability, with no other manifestations (except for those related to the reported secondary effects of the treatment). Wide base persist, and controlled MRI in February 2022 showed the same lesion as initial.

## Discussion

### ECD

To our knowledge, this case of ECD is the first to present solely with gait disorder symptoms, with central positional nystagmus (CPN) that led to the discovery of a static cerebellar syndrome (isolated wide base).

ECD is a rare disease that usually appears in people aged 40 to 70 years, with a slight male predominance (1). The symptoms depend on the specific organ or system involved.

Symmetric osteosclerosis of femur, tibia, and fibula metadiaphysis is almost always discovered on radiographs in ECD (>95% of cases). This anomaly is best depicted by the absorption of a radiotracer in ^99m^Tc bone scintigraphy or ^18^F DFG-PET/CT scan (2). Bone pain is the most common presenting symptom of ECD, and occurs in 50% of the cases. It is mainly localized in the lower limbs (1). Our patient had no complaint of pain, but the PET identified multifocal bone radiotracer uptake, predominantly at the distal half of the femur.

ECD mass formations have been reported in various tissues, such as the retroperitoneum, neck, orbit and breast (4). Intra-orbital infiltration causing exophthalmos is observed in 25% of cases. For our patient (no clinical exophthalmos), there was an infiltrate around the two optic nerves, and the patient reported visual problems only after being informed of the presence of intra-orbital masses. The ophthalmological assessment found no impact on the optic nerves, nor any abnormalities in the visual field or OCT. Radiologists and ophthalmologists should be aware of this type of abnormality, because although this presentation is rare, it should be considered in the diagnosis of ECD (5, 6).

Dense infiltration of perinephric adipose tissue, described as “hairy kidney” is also common (68% of cases) (2), and was unilateral in our patient.

Neurological signs are reported at the time of diagnosis in 25% of cases and in 50% during progression. Pituitary impairment which causes diabetes insipidus (DI) is also common (22 to 47% of cases), as well as cerebellar or pyramidal impairment, found in 25% of cases. Neuropsychiatric symptoms are reported in up to 21% of cases, and cardiovascular manifestations in 22–36% of cases. Retroperitoneal fibroinflammatory lesions affects 30% of cases, ureteral obstruction being the most frequent complication. In addition, 10% of the patients have myeloid disease, in particular myelomonocytic leukemia (1).

Ataxia is found in 23% of patients and is associated with lesions involving retrobulbar fat or optic chiasma, and the cerebellum or the dentate area. Ataxic patients frequently have dysmetria, nystagmus, Babinski sign, and/or tendon hyperreflexia on physical examination (1). In a large series with 53 patients, Parks et al. described neurological symptoms found in 47% of the cases (25/53), with imbalance (*n* = 17), headache (*n* = 11), cognitive changes (*n* = 6), dysarthria (*n* = 6), diplopia (*n* = 5), dysphagia (*n* = 4), focal sensory deficit (*n* = 4) and focal motor deficit (*n* = 2) (7). Therefore, balance disorders are frequently found in patients with ECD. However, they are poorly described, including when there is a possibility of nystagmus, which is hardly described in the literature. The study by Batjia et al. on 30 patients, reported 63% of ataxia, but without details on physical examination and no description of possible nystagmus (8). Lauricella et al., described a case with gaze nystagmus, associated with other neurological signs, including hypermetria and dysarthria (9). Na et al., reported the case of a patient with dysarthria, dysmetria and gaze nystagmus, associated with joint pain and painful axillary masses (4). Our patient presented with only CPN that was isolated and wide based. CPN has been associated with lesions involving the cerebellar vermis, nodulus and/or uvula, the dorsolateral region of the 4th ventricle, the tonsils, nucleus prepositus hypoglossi, and inferior, middle and superior cerebellar peduncles (10). The central positional nystagmus, either apogeotropic or geotropic, has been well described in inferior cerebellar peduncle (ICP) lesions damaging the fibers running from the nodulus/uvula onto the vestibular nucleus via ICP. Perverted head shaking nystagmus in this case also support an impairment of this connection or of the nodulus/uvula itself (11). ICP lesions are known to cause a distinct vestibular syndrome (12). However, on MRI, our patient had no visible lesion in the ICP. MRI showing T2 hyperintensities in the pons and middle cerebellar peduncles (MCPs), with no extension to the caudal dorsolateral portion of the 4th ventricle, which is the localization of the ICP. Our patient had lesions involving the middle cerebellar peduncles which could explain part of these presentation.

### The Central Positional Nystagmus

Due to the non-specific clinical presentation, ECD is often difficult to diagnose, and a few cases involve isolated balance disorder as a mode of discovery. Our patient had very few clinical signs, but the presence of CPN led to encephalic imaging, and thus enabled the discovery of typical intra-orbital masses, associated with T2 hyperintensities of the pons and middle cerebellar peduncles, which led to a suspicion of ECD.

PN is defined as nystagmus caused by a change in position, with respect to gravity (13). It is then classified according to various characteristics and the site of the lesion as central or peripheral PN. There are multiple characteristics whereby a distinction can be made between a central and a peripheral PN, including whether it is paroxysmal or persistent, the latency, duration, fatigability, and the response to repositioning maneuvers. Many articles are available in the literature on CPN, and a 2017 meta-analysis analyzed the characteristics of nystagmus that led to the diagnosis of CPN. They show that although no rigorous study had evaluated the prevalence of CPN, it could be related to 11–12% of the positional nystagmus seen in oto-neurology consultation (14). Given the nature of the underlying impairment which is often serious, it is important to bear in mind the clinical elements that should evoke a central vs. peripheral etiology in this context. Some of the clinical features of a CPN origin are the pureness of the nystagmus (no torsional component), or a mix of horizontal and torsional nystagmus, as well as vertical components. For nystagmus triggered by canal stimulation, the atypical character of the nystagmus as well as the absence of improvement with repositioning maneuvers is a strong argument for CPN. The absence of latency is also a reliable and frequent indicator, as was the case in our patient. So there are many arguments, and it should be borne in mind that PN is not always peripheral (14). Once again, the example of our patient illustrates the value of rigorous analysis in the presence of PN.

## Conclusion

Our case of ECD is the first that concerns a patient with only a complaint of instability, and offers a precise description of vestibulo-ocular abnormalities in this setting. A clinical examination identified CPN, and an associated static cerebellar syndrome. Together with suggestive imaging, these manifestations made it possible to diagnose ECD. Gait disturbance might be an underestimated entry sign of ECD. Therefore, the ability to differentiate CPN from peripheral PN might be crucial for early discovery of some complex neurological diseases.

## Data Availability Statement

The original contributions presented in the study are included in the article/supplementary material, further inquiries can be directed to the corresponding author.

## Ethics Statement

The studies involving human participants were reviewed and approved by Toulouse University Hospital. The patients/participants provided their written informed consent to participate in this study.

## Author Contributions

AW had the idea of case reporting, analyzed the case, and drafted the manuscript to provide intellectual content. YG revised the figures and critically reviewed the manuscript. RD, BE, LT, FV, and DC critically reviewed the manuscript. DB prepared the MRI scans for figures and critically reviewed the manuscript. FB critically reviewed the manuscript and revised the MRI sequences for interpretation. All authors contributed to the article and approved the submitted version.

## Conflict of Interest

The authors declare that the research was conducted in the absence of any commercial or financial relationships that could be construed as a potential conflict of interest.

## Publisher's Note

All claims expressed in this article are solely those of the authors and do not necessarily represent those of their affiliated organizations, or those of the publisher, the editors and the reviewers. Any product that may be evaluated in this article, or claim that may be made by its manufacturer, is not guaranteed or endorsed by the publisher.

## Abbreviations

BPPV: benign paroxysmal positional vertigo
CT: computed tomography
CPN: central positional nystagmus
MRI: magnetic resonance imaging
ECD: Erdheim Chester disease
ICP: inferior cerebellar peduncle
MAPK: mitogen-activated protein kinase
OCT: optical coherence tomography
PN: positional nystagmus
PET: positron emission tomography.
